# Mutation pattern analysis reveals polygenic mini-drivers associated with relapse after surgery in lung adenocarcinoma

**DOI:** 10.1038/s41598-018-33276-3

**Published:** 2018-10-04

**Authors:** Laura Bennett, Matthew Howell, Danish Memon, Chris Smowton, Cong Zhou, Crispin J. Miller

**Affiliations:** 1 RNA Biology Group, CRUK Manchester Institute, The University of Manchester, Alderley Park, Manchester, SK10 4TG UK; 2 Cancer Research UK Lung Cancer Centre of Excellence, The University of Manchester, Alderley Park, Manchester, SK10 4TG UK; 3 Scientific Computing Team, CRUK Manchester Institute, The University of Manchester, Alderley Park, Manchester, SK10 4TG UK; 4 Division of Cancer Sciences, Faculty of Biology, Medicine and Health, Manchester Cancer Research Centre, University of Manchester, Wilmslow Road, Manchester, M20 4GJ UK; 5 Present Address: Cancer Research UK Cambridge Institute, University of Cambridge, Li Ka Shing Centre, Robinson Way, Cambridge, CB2 0RE UK

## Abstract

The genomic lesions found in malignant tumours exhibit a striking degree of heterogeneity. Many tumours lack a known driver mutation, and their genetic basis is unclear. By mapping the somatic mutations identified in primary lung adenocarcinomas onto an independent coexpression network derived from normal tissue, we identify a critical gene network enriched for metastasis-associated genes. While individual genes within this module were rarely mutated, a significant accumulation of mutations within this geneset was predictive of relapse in lung cancer patients that have undergone surgery. Since it is the density of mutations within this module that is informative, rather than the status of any individual gene, these data are in keeping with a ‘mini-driver’ model of tumorigenesis in which multiple mutations, each with a weak effect, combine to form a polygenic driver with sufficient power to significantly alter cell behaviour and ultimately patient outcome. These polygenic mini-drivers therefore provide a means by which heterogeneous mutation patterns can generate the consistent hallmark changes in phenotype observed across tumours.

## Introduction

Large-scale cancer sequencing projects have led to a rapid expansion in the catalogue of putative driver mutations and revealed striking genetic heterogeneity across the sets of mutations identified in different patients^1^. While a small number of genes are frequently mutated at rates >20%, the majority occur at low frequencies^2^. In lung adenocarcinoma (LUAD), for example, the most frequently mutated gene, *TP53*, is altered in only ~50% of patients^3^. Despite this extensive heterogeneity, cancer is characterized by a set of ‘hallmark’ changes in phenotype^4,5^, even in the absence of a known driver mutation that can be linked directly to one of these canonical processes^2^. A critical question, therefore, is how these consistent phenotypes emerge from such underlying genetic diversity, and the task of identifying the mutations that contribute to these changes remains challenging^6^.

One possibility, as hypothesized by Castro-Giner *et al*.^7^, is that in tumours lacking a clear major driver, oncogenic transformation may instead occur through the collaborative action of a set of ‘mini-drivers’, each with a weak individual effect. In this model, these mini-driver mutations accumulate to form a polygenic driver with sufficient power to lead to tumour promoting effects and phenotypic changes^7,8^. Polygenic mini-drivers may also function in combination with conventional driver mutations. This model of the disease is in keeping with findings from large scale Genome Wide Association Studies (GWAS) that have shown, for typical traits, that even the most significant loci explain only a fraction of the predicted genetic variation^9^; the remaining “missing heritability” is provided by the contribution of multiple Single Nucleotide Polymorphisms (SNPs) that each fall below the threshold for genome-wide significance^9^. While it is tempting to speculate that cancer genes may behave similarly, there has been little experimental evidence so far to support this polygenic ‘mini-driver’ model of oncogenesis.

One of the challenges with validating such a model arises from the need to consider mutations with weak effects. Current approaches that attempt to predict driver mutations from mutation frequency do so by seeking mutations significantly more abundant than the estimated background mutation rate^2^. Since the background rate is dependent on multiple factors including genomic position, the mutation load in an individual patient, and gene size, these factors are represented as additional covariates in the numerical model^1,2^. This tends to raise the bar for long genes and/or those located in regions with higher mutation frequencies. Overall these techniques successfully remove multiple artefacts from candidate driver lists, including false positives such as *TTN*, which is extremely large, or olfactory receptors that are not expressed in the tissue of interest^1^.

While these models have significantly advanced the state of the art, they are focused on finding conventional drivers with significant individual effect. In the context of a mini-driver model, where individual components of a polygenic driver are each expected to have a minimal effect, these genes would not be expected to pass the threshold for overall significance. Similarly, approaches that aim to predict high impact changes to the encoded protein sequence (e.g.^10,11^) will tend to ignore the weak-effect mutations of most interest when searching for polygenic drivers. Thus, taken together, these data suggest that alternative mathematical models might prove useful when seeking to expand the catalogue of driver mutations to include polygenic drivers.

Here, we use Complex Network analysis to test the ‘mini-driver’ model proposed by Castro-Giner and colleagues^7^. We do this by building models that integrate somatic mutations with gene-expression data from LUAD patients. Complex networks are frequently used to represent natural systems. They encompass fields as diverse as social network analysis^12^, epidemiology^13^, biology^14^ and computing^15^. Complex networks exhibit community structure: a modular organization comprising discrete, highly connected sets of nodes with sparse interconnections^16,17^. Irrespective of the domain of interest, nodes within the same module, or ‘community’, typically share functionality^14,18^. They thus offer a powerful framework with which to explore interactions within systems and to identify common patterns of activity within heterogeneous populations^19^. Gene regulatory interactions also lend themselves well to complex network models, since co-expression is often indicative of common patterns of regulatory control^20,21^. This results in a modular structure in which functionally related genes congregate as sets of densely interconnected nodes within the network^9,16^.

Since cancers exhibit a set of consistent hallmark phenotypes, irrespective of their underlying mutation profiles^4^, we speculated that these might sometimes arise through functional changes resulting from the accumulation of multiple weak insults to the same cellular subsystems. We therefore hypothesized that if present, polygenic mini-driver mutations would tend to occur within sets of functionally related genes, and thus congregate within specific modules in a co-expression network.

**Figure 1:**
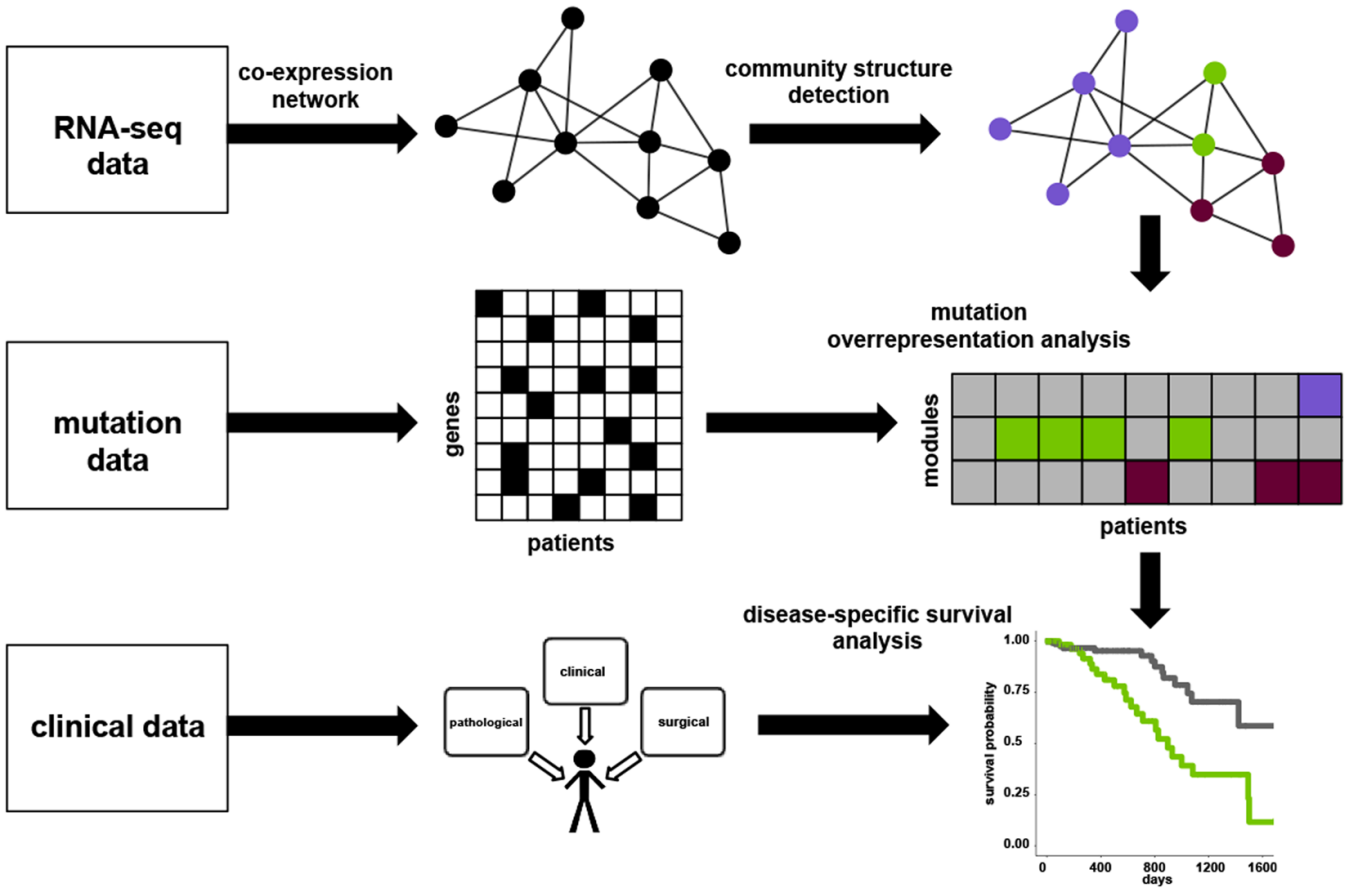
Overall strategy. Co-expression networks were generated from RNA-seq data. Community structure detection was applied to partition the networks into modules (purple, green and maroon nodes). For each patient, genes with non-synonymous mutations were enumerated, then mapped to the network modules, and overrepresentation analysis was performed. Modules with (purple, green and maroon cells), or without (grey cells) significant enrichment for mutations were identified. Disease-specific survival analysis was then conducted.

We sought to test this hypothesis as follows (summarized in Fig. 1): We first built independent co-expression networks representing LUAD tumour and matched normal samples. We segmented these networks using the Louvain greedy optimization algorithm^22^. We then mapped mutations from an independent cohort of LUAD patients onto these modular networks. Rather than assessing the significance of each individual mutation, as would be appropriate when seeking high impact single gene drivers, we instead use complex networks to model potential relationships between genes. We then seek regions of the networks that are significantly enriched for mutations. In this way we consider statistical significance in terms of the aggregate behaviour of multiple mutations, rather than on an individual gene basis. This makes it possible to consider genes that would otherwise be dismissed using conventional ranking algorithms because their individual effect is too small to raise them above the score threshold for individual significance.

Finally, we validated the approach by assessing the biological relevance of these aggregate gene sets by asking whether they were associated with patient outcome. This allowed us to derive a polygenic mutation pattern predictive of disease-specific survival, thus confirming the biological relevance of the signature.

## Results

We enumerated all non-synonymous mutations across 660 LUAD samples^23^. Mutation frequency distributions for both genes and residues exhibited little overlap between tumours (Supplementary Fig. 1). Only 11 protein-coding genes were mutated in more than 25% of tumours, and only one, *TP53*, harboured a mutation in more than 50% of samples (347/660; Supplementary Fig. 1a). At the residue level, while canonical mutations such as *KRAS*^G12^, *EGFR*^L858R^, *TP53*^R158P^ and *BRAF*^V600E^ were detected, no individual non-synonymous Single Nucleotide Variants (SNVs) occurred in more than 16% of tumours (101/660; Supplementary Fig. 1b). Thus, in keeping with previous reports^1^, these patients exhibited a high degree of heterogeneity between their individual mutation spectra.

### Lung cancer co-expression network exhibits a higher degree of modular structure

**Figure 2:**
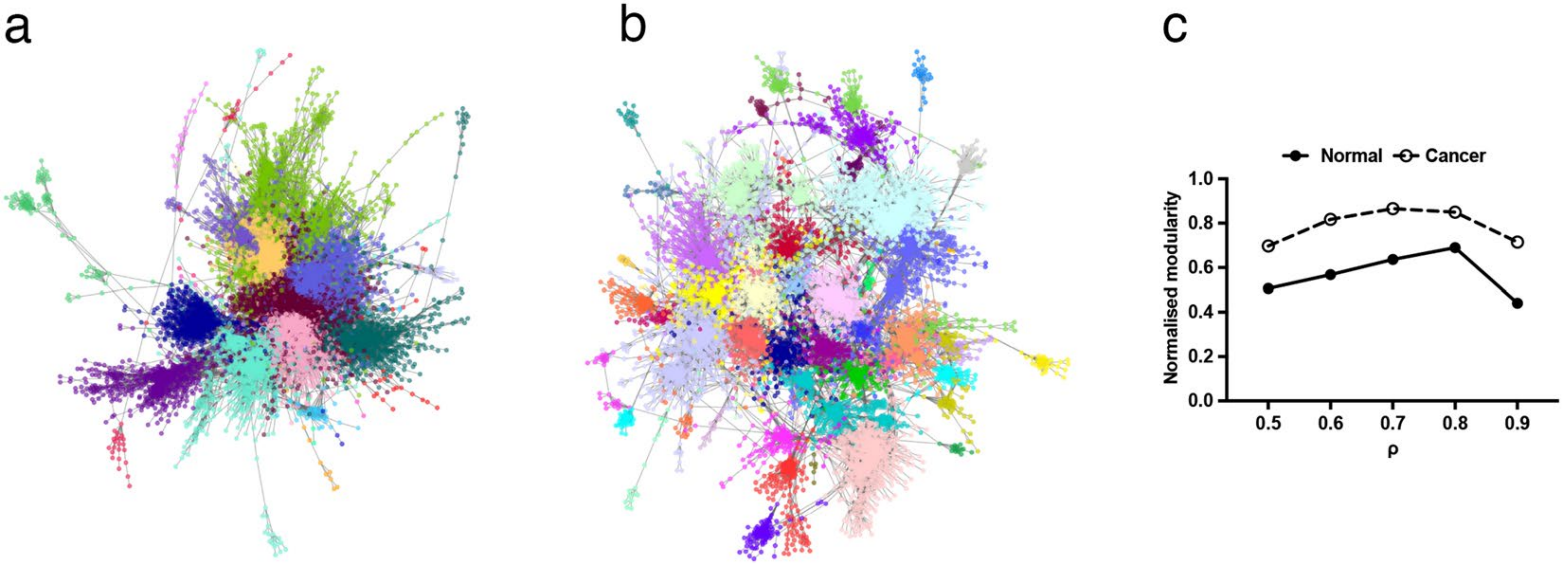
Network visualization and community structure detection. (**a**) Normal tissue derived co-expression network. Edges included at *ρ* ≥ 0.8. Networks were visualised using the Cytoscape(49) perfuse force-directed layout algorithm, without knowledge of the identified community structure. Each community was assigned a colour according to the partitions identified by the Louvain algorithm. (**b**) Cancer tissue derived co-expression networks. All parameters as a. (**c**) Normalised modularity values for normal and tumour derived networks at different values of *ρ*.

Co-expression networks were then derived from a dataset comprising 58 LUAD matched tumour normal pairs^24^. Tumour and normal samples were treated independently, using Pearson correlation, *ρ*, as the underlying distance metric, since in this context it outperforms other more complex mathematical relationships^25^. Co-expression networks were constructed with threshold values, *ρ* = 0.5, 0.6, 0.7, 0.8 and 0.9 (network properties summarized in Supplementary Table 1). Modularity quantifies the strength of community structure exhibited by a network^16^. The Louvain greedy modularity optimization algorithm^22^ was used to partition the network into modules, thus providing an unbiased definition of individual communities within the tumour and normal networks (Fig. 2a and b; gene-module memberships for *ρ* = 0.8 given in Supplementary Table 2). These data revealed a striking increase in normalized modularity with respect to matched normal samples, irrespective of the Pearson cut off (Fig. 2c). Since these organizational changes were consistent at multiple scales, all subsequent data are presented at *ρ* = 0.8.

Two additional global metrics were also considered: Network Density, and Average Path Length. Network density refers to the number of edges in a network scaled by the maximum number of possible edges, while average path length is computed as the mean of the shortest paths between all pairs of nodes in the network. Both measures indicate the degree of connectivity of a network. In keeping with their increased modularity, the cancer networks also exhibited reduced density (normal = 0.0092, cancer = 0.0049) and increased average path length (normal = 5.24, cancer = 8.09). Together, these data demonstrate a system-wide loss of connective elements from the normal networks. We therefore reasoned that the network generated from the matched normal material, which includes elements perturbed or lost following oncogenic transformation, would provide a better baseline for understanding how cancer disturbs the normal functioning of the cell^26^. This is particularly important in the context of tumours, because many mutations result in transcripts being lost from the cancer-derived coexpression network, either through altered patterns of expression and loss of overall correlation, or through the creation of truncated or rapidly degraded transcripts^27^ that are no longer detected by sequencing. Many mutated genes, including tumour suppressors^27^, are consequently not represented in the cancer networks.

We then used the modular architecture of the normal network as a map onto which to project the mutational profiles from individual LUAD patients.

### The normal network acts as a unifying framework for heterogeneous LUAD patients

We generated the mutational spectra for 602 LUAD patients^23^ by enumerating, for each sample, all genes with a non-synonymous somatic mutation. These samples were independent from those used to derive the expression networks. We then mapped the mutation profile for each patient onto the baseline normal network and performed a mutation overrepresentation analysis, seeking modules with a disproportionate number of mutations. Importantly, we did not filter mutations using existing models of significance focused on single-gene drivers, since the goal was to consider genetic lesions with weak effects that would be expected to fall at or below their significance threshold. Instead, we estimated statistical significance by using the normal networks to provide an unbiased grouping of genes into discrete subsystems, and then testing whether regions of the network were consistently enriched for mutations across the patient cohort.

**Figure 3:**
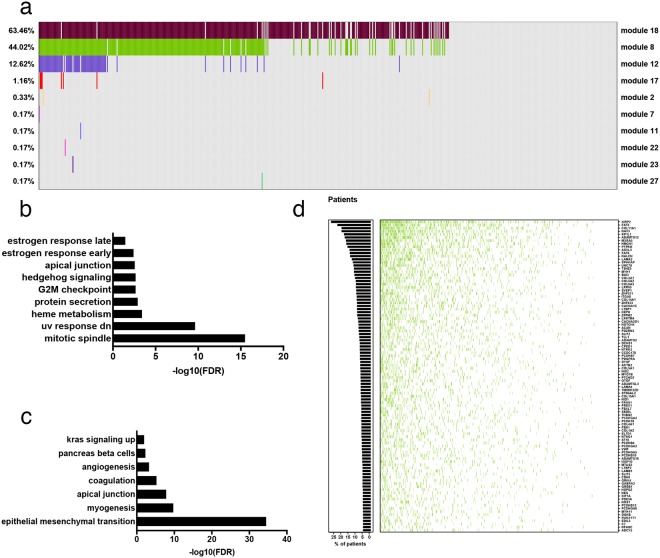
Mutation overrepresentation analysis. (**a**) Modules with a statistically significant number of mutated genes in the normal network (non-grey cells). Module number is indicated on the right hand side of the figure, overall representation within the cohort on the left. Colours correspond to the modules in Fig. 2a. Only modules enriched for mutations in at least one patient are shown. (**b**) Broad Institute Hallmark genesets enriched in module 18. (**c**) Broad Institute Hallmark genesets enriched in module 8. (**d**) The top 100 most frequently mutated genes in module 8. Each row corresponds to a gene, columns, patients. Green cells indicate that a gene is mutated in the corresponding patient. The histogram shows the percentage of patients with at least one mutation in each gene.

Since the majority of mutated genes would be expected to harbour only passenger mutations^28^ that confer no proliferative or survival advantage, a reasonable *a priori* expectation would be for them to be randomly distributed across the network. Instead, we observed a pattern in which specific network modules were frequently enriched for mutations relative to the overall mutational load for that patient (FDR < 0.05). In particular, two modules in the normal networks repeatedly exhibited a disproportionate accumulation of mutations in 63% and 44% of patients respectively (modules 18, 8; Fig. 3a). Importantly, only a weak pattern of enrichment was observed in the tumour-derived network (Supplementary Fig. 2a), demonstrating the additional analytical power lent by projecting the mutation data onto the normal network.

### The majority of tumours have a significant overrepresentation of mutations in modules enriched for mitotic spindle or metastasis associated genes

In total 71% of samples were enriched for mutations in either module 18 or 8. The most frequently enriched module (module 18) comprised a significant number of genes important in mitotic spindle assembly and the G2 DNA damage checkpoint (Broad Institute Hallmark genesets shown in Fig. 3b; top 20 gProfileR results in Supplementary Fig. 2b and full results in Supplementary Table 3). This module included several of the canonical oncogenes and tumour suppressors (as defined in^24^; *EGFR*, *ERBB2*, *PIK3CA*, *NF1*, and *RB1*).

By contrast, module 8 comprised genes associated with extracellular matrix organization, epithelial-mesenchymal transition, *TGFβ* receptor signalling pathway, collagen metabolism, blood vessel development, blood circulation, and angiogenesis (Broad Institute Hallmark genesets shown in Fig. 3c; top 20 gProfileR results in Supplementary Fig. 2c; full results in Supplementary Table 3). These are all processes involved in tumour dissemination, the establishment of metastases and, ultimately, poorer prognosis^29^.

Importantly, while the most frequently mutated gene in module 8, *XIRP2*, is mutated in 27% of patients, and only 24 genes mutated in at least 10% of tumours, the majority of genes in the module are altered only in a small proportion of cases (Fig. 3d; similarly for module 18, Supplementary Fig. 2d). Thus, viewing these heterogeneous samples in the context of the modular landscape of the normal network reveals unifying patterns not evident at the level of individual genes.

While expression-based signatures associated with metastasis have been proposed, a mutation-based signature of metastasis remains elusive^28^. This mutation pattern, which occurs within a network module associated with tumour progression and metastasis, was therefore of substantial interest. We next asked whether the frequent enrichment of mutations within this module might provide evidence in support of a mini-driver model of cancer, reasoning that if the accumulation of mutations within module 8 was indeed biologically relevant, it would correlate with patient characteristics. Since metastasis is the most common cause of cancer-death^28^, we focused on disease specific survival.

### Accumulation of mutations within the metastasis-associated module predicts worse 5-year disease-specific survival

**Figure 4:**
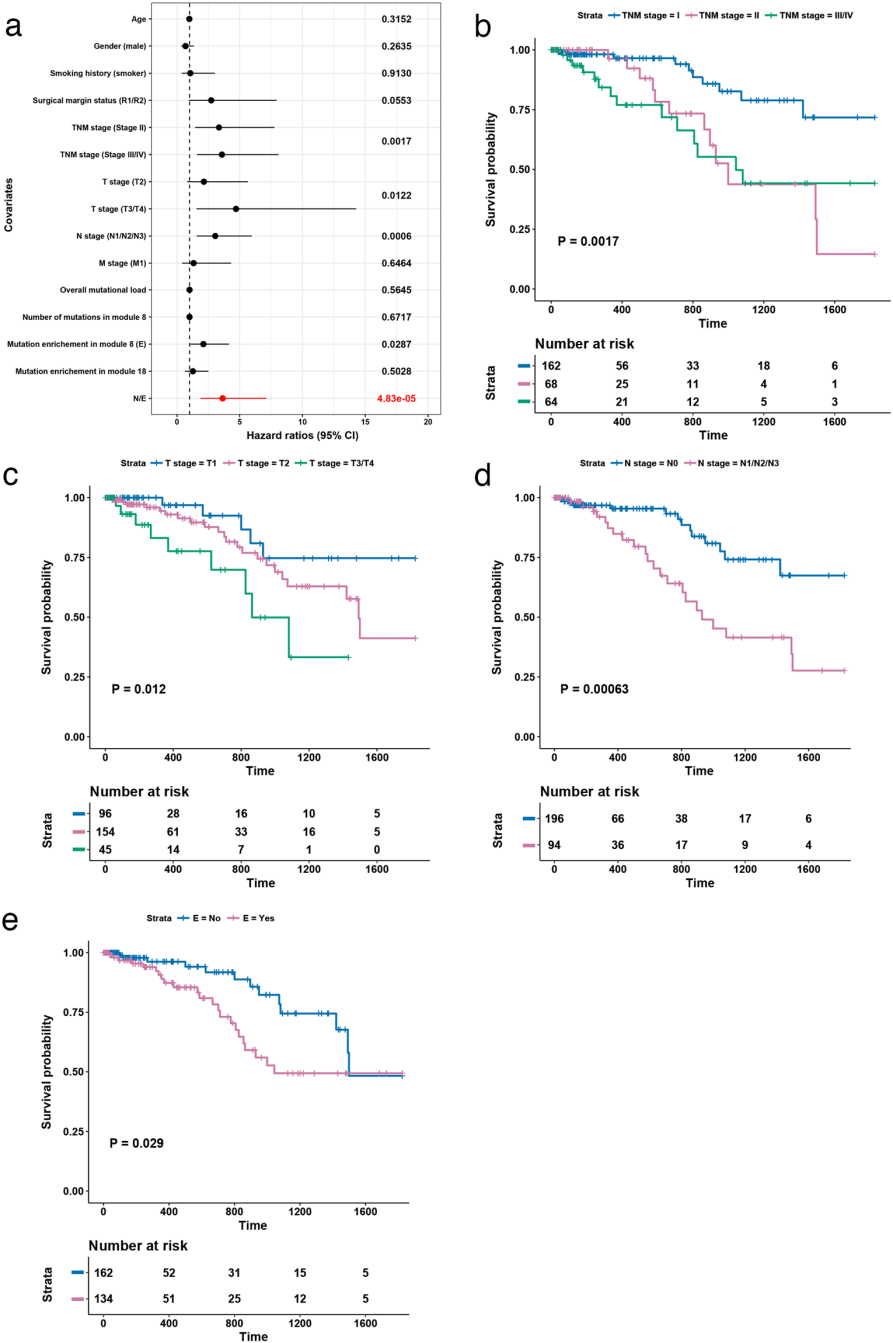
Five-year disease-specific survival. (**a**) Forest plot with hazard ratios from the univariate Cox proportional hazards regression analysis. Significance values are the global model log-rank p-values. E status in red. Kaplan-Meier curves with log-rank p-value for (**b**) TNM stage, (**c**) T stage, (**d**) N stage, (**e)**, E status (E = “Yes” indicates a significant number of mutations in module 8).

Clinical characteristics^23,30^, including known independent prognostic factors, were available for a subset of the LUAD cohort (296 patients; Table 1). A univariate Cox proportional hazards regression analysis was used to determine potential factors contributing towards di sease-specific survival, including overrepresentation of mutations in modules 8 and 18 (Fig. 4a and Supplementary Table 4). TNM stage^31^, T stage (T; tumour size), N stage (N; degree of spread of cancer to the lymph nodes) and Enrichment of mutations in module 8 (‘E status’) had a significant negative correlation with survival (P < 0.05; Kaplan-Meier curves in Fig. 4b–e).

**Table 1:** Summary of clinical features of 296 LUAD patients.

**Age**, **yr**	
Median (IQR)	66 (59–72)
**Gender (%)**	
Male	139 (47)
Female	157 (53)
**TNM stage (%)**	
Stage I	162 (54.7)
Stage II	68 (23)
Stage III	47 (15.9)
Stage IV	17 (5.7)
Unknown	2 (0.7)
**T stage (%)**	
T1	96 (32.4)
T2	154 (52)
T3	31 (10.5)
T4	14 (4.7)
TX	1 (0.3)
**M stage (%)**	
M0	200 (67.6)
M1	16 (5.4)
MX	77 (26)
Unknown	3 (1)
**N stage (%)**	
N0	196 (66.2)
N1	53 (17.9)
N2	40 (13.5)
N3	1 (0.3)
NX	5 (1.7)
Unknown	1 (0.3)
**Surgical margin status (%)**	
R0	198 (67)
R1	9 (3)
R2	1 (0.3)
RX	13 (4.4)
Unknown	75 (25.3)
**Smoking history**	
Smoker	240 (81)
Never	49 (17)
Unknown	7 (2)
**Overall mutational load**	
Median (IQR)	192.5 (90.75, 378)
**Number of mutations in module 8**	
Median (IQR)	16 (6, 32)
Total no. of deaths	45
No. of deaths with tumour	36

While M stage (M; spread of cancer to another part of the body) is a known prognostic factor^31^, it was not associated with survival in this analysis. This is a consequence of the high proportion of patients without metastatic disease (M0 = 68%, M unknown = 27%). Further, 27% of M-unknown patients are highly likely to represent M0 disease, as the TCGA dataset is comprised almost exclusively of samples collected at the time of curative intent surgery, which is not routinely used as a treatment for metastatic (M1) disease.

Importantly, a significant number of mutations in module 8 (i.e. E = “yes”) was negatively associated with disease-specific survival (P = 0.0287, HR = 2.1, CI: 1.1–4.2; Fig. 4e, Supplementary Table 4), confirming the prognostic significance, and therefore biological relevance, of the mini-driver pattern. No such correlation was found for module 18. Given the core cell cycle role of genes within this module, it may be that mutations here are indicative solely of the presence of a tumour, rather than of its aggressiveness.

Overall mutational load was not associated with survival (Supplementary Table 4), establishing that the signature was not simply a proxy for genome-wide mutation rates. Furthermore, while enrichment for mutations in module 8 was associated with survival, absolute mutation count within the module was not (Supplementary Table 4). This may be because when mutation rates are high throughout the cell, rather than simply in module 8, there is a general loss of fitness, reducing cell survival, and thus making relapse less likely. Alternatively, these cells may present better targets to the immune system^32^.

**Figure 5:**
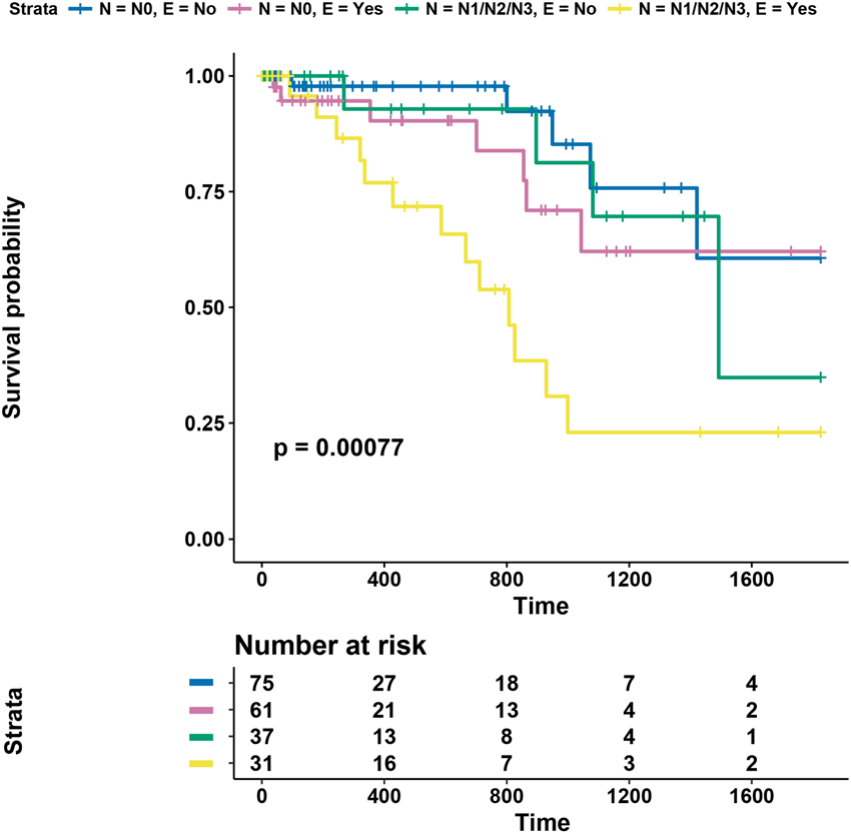
Multivariate model combining nodal status and module 8 enrichment. Five-year disease-specific survival. Kaplan-Meier curve with log-rank p-value for multivariate model incorporating N and E status (E = ‘yes’ indicates significant enrichment of mutations in module 8).

We next performed a multivariate analysis considering all covariates from the univariate analysis with P < 0.2. At this threshold, surgical margin status (R; degree of residual tumour after surgery) was also included in the initial model, in addition to TNM stage, T stage, N stage, and E status, as before. Backward stepwise selection was then used to identify the best model, with goodness of fit determined using the AIC score. Data with missing values were removed, leaving 204 patients in the analysis. The final model included N stage and enrichment of mutations in module 8 (E status), with AIC = 219.5 (Fig. 5). Taken together, these data thus demonstrate the prognostic value of E status in patients with nodal disease.

### Enrichment of mutations in the metastasis module predicts worse 5-year disease-specific survival in patients with N2 lymph node disease

**Figure 6:**
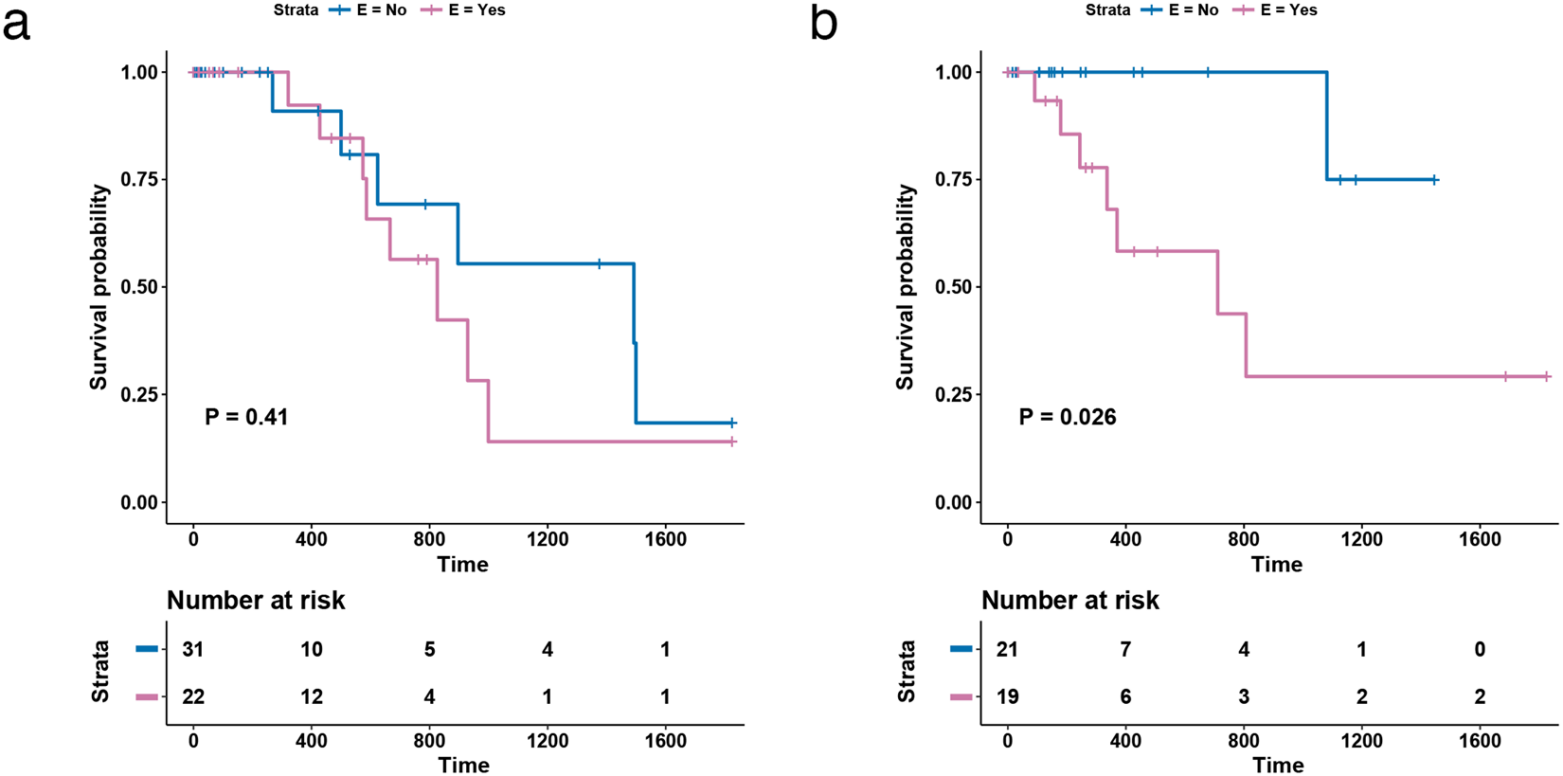
5-year disease-specific survival results for N-stage patients. Kaplan-Meier curves with log-rank p-value for patients with N stage equal to (**a**) N1 and (**b**) N2, stratified by E status.

We further investigated the subset of patients with lymph node metastases. Lymph node involvement is an important prognostic factor; with increasing N stage, cure rate following surgery decreases^33^. We inspected N1 and N2 patients individually and stratified samples based on E status (there was only a single N3 patient; Fig. 6a,b). In patients with N1 disease, E status was not associated with survival (P = 0.4129, HR = 1.6, CI: 0.5–4.6; Fig. 6a). In patients with N2 involvement, E status significantly partitioned samples based on 5-year disease-specific survival (P = 0.0264; Fig. 6b), with only one cancer-specific death in patients with negative E status. However, due to the small sample size of N2 patients, there is a large 95% confidence interval for the hazard ratio (HR = 7.7, CI: 0.9–62.9). Therefore, while this result requires further validation, it indicates that N2 patients with an accumulation of mutations in the metastasis module are more likely to relapse and die with tumour following surgery.

These data are of clinical interest because the optimum treatment strategy for Stage IIIA N2 disease is unclear^34^. Dependent on clinical characteristics, primary treatment may involve surgery or chemoradiotherapy. In those patients who undergo surgery, the benefit of postoperative radiotherapy (PORT) is uncertain and current practice is to consider this adjuvant approach based on an individual assessment of relapse risk. Our data reveal a set of genes associated with poor outcome, with the potential to stratify postoperative Stage IIIA N2 patients for a prospective clinical trial of PORT efficacy in this setting.

## Discussion

By building network models representing the expression profiles of normal samples, and then mapping the mutation spectra of individual lung cancer patients onto these networks, we were able to place somatic mutations into the context of lung tissue before the system-wide reorganization of gene expression that occurs during oncogenesis. This allowed us to identify a set of genes with a mutation pattern predictive of disease specific survival that, in combination with nodal status (N-stage), led to an improved classifier. A significant number of genes within this set were associated with the ECM, EMT, tumour dissemination, and metastasis (Fig. 3c and Supplementary Fig. 2c), in keeping with its significance in patients who relapse following surgery. Further, the enrichment for mutations within this gene set has the potential to better stratify patients with N2 nodal disease.

While 44% of tumours harboured a statistically significant number of mutations within the ECM module (module 8), individual genes were mutated at much lower rates (Fig. 3d), in keeping with a ‘mini-driver’ model in which it is the polygenic combination of multiple genes within the module that leads to the observed effect. We also detected substantial enrichment in the network module associated with the mitotic spindle and DNA damage repair (module 18; Fig. 3a and b and Supplementary Fig. 2b). This was observed for 84% of patients for which the metastatic module was enriched, and in over half (63%) of all patients studied.

These data also demonstrate the utility of alternative models for assessing the significance of mutations. Recently Waks and colleagues were able to identify a set of candidate tumour suppressors encoding extremely long chromatin regulators^35^. They achieved this in part by relaxing gene-length constraints in favour of additional functional annotation data. Here we make use of the extra structure provided by co-expression networks to find significant sets of mutations.

The complex network approach of integrating expression and somatic mutation data also demonstrates the importance of looking beyond a binary driver versus passenger view of cancer and to integrate the concept of mini-drivers into models of tumorigenesis. Importantly, mapping mutations onto expression data derived from tumour samples did not yield similar patterns (including prognostic significance; data not shown), thus highlighting the utility of using normal samples to provide a reference framework. Our approach therefore differs from previous studies, which have tended to focus on the gene expression profiles derived from tumour cells, either to develop expression-based signatures, e.g.^36–40^, or to consider them in combination with somatic mutation profiles^41^.

Taken together, these data therefore provide significant evidence in support of the mini-driver models of cancer proposed by Castro-Giner and colleagues^7^, and a potential explanation of how the same hallmark phenotypes can emerge in different tumours with a high degree of heterogeneity. This has significant implications for therapeutic strategies, since monogenic approaches that target an individual driver gene are unlikely to be beneficial in the context of a tumour driven by a set of collaborating mini-drivers. Instead, for these tumours, multi-target and pathway focused strategies that aim to treat cellular subsystems, rather than a gene, are more likely to be effective.

## Methods

### RNA-seq data

The Cancer Genome Atlas (TCGA) LUAD expression dataset comprised 116 matched-normal lung adenocarcinoma samples (58 normal and 58 cancer)^24^. Raw data for were aligned using MapSplice^42^ against human reference genome hg19 (minimal filtering) and read counts were generated at the exon level (Ensembl version 70) and transformed into RPKM values.

### Co-expression network construction and analysis

The expression datasets of normal and cancer samples were analysed separately. Genes in the 40th percentile of summed RPKM values were discarded due to low levels of expression. Pearson correlations were calculated between the remaining gene expression profiles, and normal and cancer co-expression networks were constructed with the following threshold values, *ρ* = 0.5, 0.6, 0.7, 0.8 and 0.9. Network analysis was carried out using the igraph package in R^43^. The greedy modularity optimization method, Louvain^22^, implemented in the ‘multilevel.community’ function, was used for community structure detection. For both co-expression networks, randomly rewired networks that preserved the original network’s degree distribution were generated repeatedly using a computationally optimized implementation of the ‘rewire.edges’ function. In order to compare modularity across networks with different size and connectivity, modularity was normalized using the randomly generated networks, as described in^44,45^.

### Mutation data

Mutation data for 1144 non-small cell lung cancer patients were downloaded from cBioPortal^23,30^. This dataset included both lung squamous and lung adenocarcinoma samples. 660 LUAD patients were selected for further analysis: 501 from the TCGA cohort^24^, and 159 from Imielinski *et al*.^3^. For each patient, non-synonymous somatic mutations were enumerated. Silent mutations were not included (Variant_Classification = ‘Silent’), while non-silent mutations (Variant_Classification equal to any of the following: Missense_Mutation, Nonsense_Mutation, Frame_Shift_Del, Frame_Shift_Ins, In_Frame_Ins, In_Frame_Del, Missense, Splice_Site, Translation_Start_Site, Nonstop_Mutation) were considered non-synonymous. Only Single Nucleotide Variants (Variant_Type = ‘SNP’) were used to generate calculate mutation frequency distributions for residues.

### Mutation overrepresentation analysis

Only 602/660 LUAD samples were considered, since 58 of the TCGA patients from the cBioPortal^30^ data were also included in the cohort used to generate the co-expression networks. For each remaining patient, their set of mutated genes was mapped onto the partitioned networks. A one-tailed Fisher’s exact test identified modules comprising a significantly disproportionate number of mutated genes relative to the background population of the entire network and the patient’s overall mutational load. P-values were adjusted using the Benjamini-Hochberg (BH) method for multiple testing to control the false discovery rate (FDR). A significance threshold of FDR < 0.05 was used.

### Functional enrichment

The gProfileR package in R^46^ was used to identify overrepresented Gene Ontology Biological Processes^47^ and additionally, Broad Institute Hallmark genesets^48^ using a hypergeometric distribution (background population: the whole network; FDR < 0.05).

### Clinical data and 5-year disease-specific survival analysis

Clinical annotation for the 660 LUAD patients were downloaded from cBioPortal^23,30^. Data corresponding to patient tumours for which gene expression data were used to generate the correlation networks were removed, leaving 602 patient samples. Disease-specific survival analysis requires the following information: ‘time’ taken as ‘days to death’ if available and ‘days to last follow-up’ otherwise, vital status, and whether the patient was with tumour or tumour free. Patients ‘with tumour’ at time of death were classified as having disease-specific death. LUAD patients from Imielinski *et al*.^3^ were not included in the survival analysis, since these data were not available. Tumour status (with tumour or tumour free) for the TCGA patients was obtained from the RTCGA package in R^49^, as was residual tumour status. A total of 296 patients had the required data and were included in the analysis.

Survival analysis was carried out using the ‘survival’ package in R^50^. Univariate and multivariate Cox proportional hazards regression analyses identified factors with significant impact on disease-specific survival. Multivariate analysis was performed by first identifying significant variables (log-rank p-value < 0.2) in a univariate analysis. These significant covariates were then used to form an initial multivariate model. Backwards stepwise regression was then performed, using the ‘stepAIC’ function from the ‘MASS’ package in R, to refine the model^51^. Log-rank p-values (P), associated hazard ratios (HR) and 95% confidence intervals (CI) were reported. Kaplan-Meier curves were generated for significant variables from the multivariate analysis.

## Electronic supplementary material

Supplementary information

Supplementary Table S1

Supplementary Table S2

Supplementary Table S3

Supplementary Table S4

## Data Availability

All data are available via TCGA.
