# Supplementary material for: Mutation pattern analysis reveals polygenic mini-drivers associated with relapse after surgery in lung adenocarcinoma
Source: Sci Rep. 2018 Oct 4;8:14830. doi: 10.1038/s41598-018-33276-3 (PMC6172282; doi:10.1038/s41598-018-33276-3)
Supplement: Supplementary file 1 — Supplementary information [file 41598_2018_33276_MOESM1_ESM.pdf]

Mutation pattern analysis reveals polygenic mini-drivers associated with relapse after surgery in lung adenocarcinoma

[1]Laura Bennett, [1,2]Matthew Howell, [1$]Danish Memon, [3]Chris Smowton, [4]Cong Zhou, *[1,2]Crispin J Miller

[1]RNA Biology Group, CRUK Manchester Institute, The University of Manchester, Alderley Park, Manchester SK10 4TG UK

[2]Cancer Research UK Lung Cancer Centre of Excellence, The University of Manchester, Alderley Park, Manchester SK10 4TG UK

[3]Scientific Computing Team, CRUK Manchester Institute, The University of Manchester, Alderley Park, Manchester SK10 4TG UK

[4]Division of Cancer Sciences, Faculty of Biology, Medicine and Health, Manchester Cancer Research Centre, University of Manchester, Wilmslow Road, Manchester, M20 4GJ UK

[$]Current Address: Cancer Research UK Cambridge Institute, University of Cambridge, Li Ka Shing Centre, Robinson Way, Cambridge, CB2 0RE, UK; European Molecular Biology Laboratory (EMBL), European Bioinformatics Institute, Wellcome Genome Campus, Hinxton, Cambridge CB10 1SD, UK

Running title: Polygenic mini-drivers in lung cancer

Keywords: lung adenocarcinoma, tumour heterogeneity, polygenic signatures, cancer genomics, complex networks, mini-drivers

***Corresponding author:** Crispin J. Miller CRUK Manchester Institute, The University of Manchester, Alderley Park, Manchester SK10 4TG UK; Phone: 44161-446-3176; Fax: 44161-446-3109; Email: crispin.miller@cruk.manchester.ac.uk

Supplementary Data

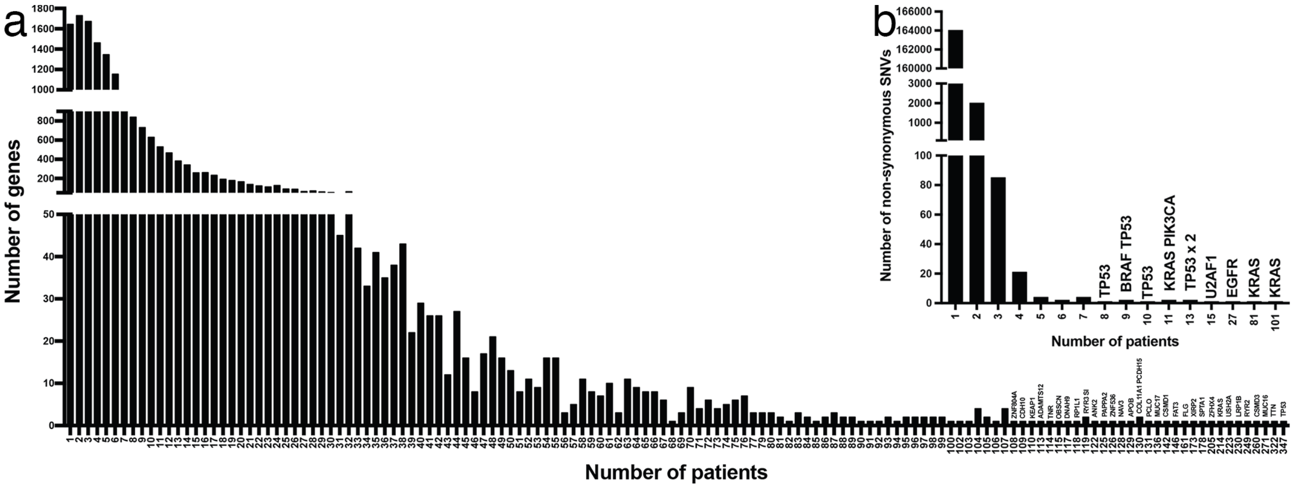

Supplementary Figure S1. Frequency distribution showing the rate of non-synonymous mutations in protein coding genes across 660 LUAD patients. **a**, summarized by gene. **b**, summarized by SNV count, gene names occur multiple times when different nucleotides are mutated in the same gene.

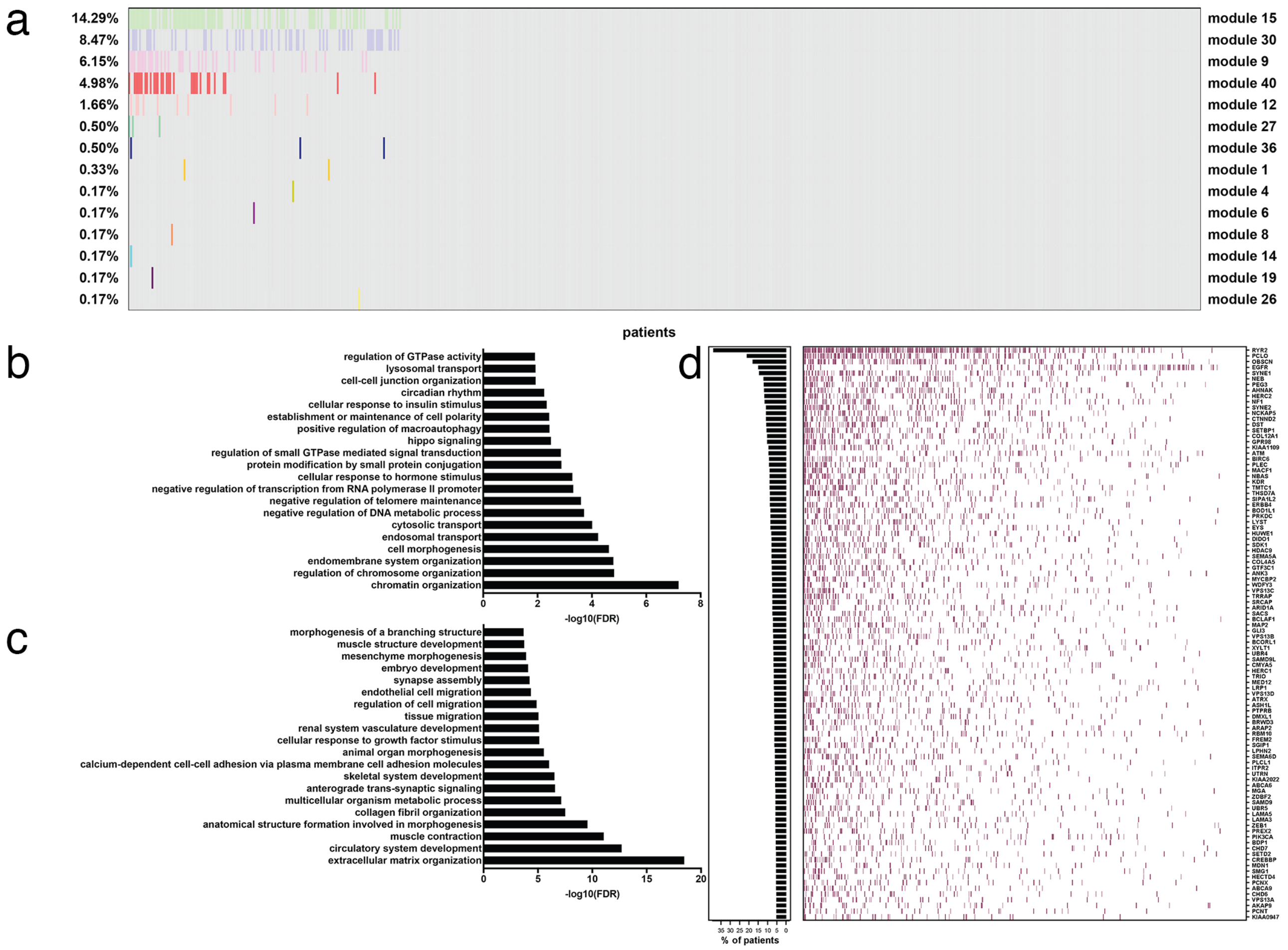

Supplementary Figure S2. a, Mutation overrepresentation analysis in the cancer network. Non-grey cells indicate that a patient has a statistically significant number of mutated genes within a module. Module number is indicated on the right hand side of the figure. Colours correspond to the modules in Fig. 2b. Only modules enriched for mutations in at least one patient are shown. **b**, Top 20 gProfileR results (Biological Processes) enriched in module 18 in the normal network. **c**, Top 20 gProfileR results (Biological Processes) enriched in module 8 in the normal network. **d**, The top 100 most frequently mutated genes in module 18 in the normal network. Each row corresponds to a gene, columns, patients. Maroon cells indicate that a gene is mutated in the corresponding patient. The histogram shows the percentage of patients with at least one mutation in each gene.

Supplementary Table S1. Networks summarized by node and edge numbers for ρ = 0.5, 0.6, 0.7, 0.8 and 0.9.

Supplementary Table S2. Gene-module membership for the normal and cancer networks at ρ = 0.8, as determined by the Louvain greedy modularity optimization method [23].

Supplementary Table S3. Full gProfiler results of Gene Ontology Biological Processes overrepresented in modules 8 and 18.

Supplementary Table S4. Univariate Cox proportional hazards regression results for covariates described in Table 1. Global statistical significance of each model is given by the log-rank p-value.
